# DISASTER AND EMERGENCY SUPPORT NETWORKS OF FAMILIES PROVIDING CARE TO PERSONS LIVING WITH DEMENTIA

**DOI:** 10.1093/geroni/igae098.0756

**Published:** 2024-12-31

**Authors:** Sato Ashida, Freda Lynn, Maria Donohoe, Emily Killian

**Affiliations:** University of Iowa, Iowa City, Iowa, United States; University of Iowa, Iowa City, Iowa, United States; University of Iowa, Iowa City, Iowa, United States; University of Iowa College of Public Health, Iowa City, Iowa, United States

## Abstract

Persons living with dementia (PLWD) and their caregivers are especially vulnerable in disaster situations. Research describes the composition and function of caregiving support networks, but not how these networks support families in terms of disaster preparedness. Currently, a study is underway to investigate disaster and emergency support networks and caregiver resilience. The baseline data collected thus far show that caregivers (N=59) identified 633 support members in total (M=10.4 members per respondent) via four prompts: family members important to PLWD (25%), others important to PLWD (37%), others important to themselves (25%), and additional members who would support them in disaster situations (12%). Of all named members, respondents identified 44% (M=4.5 members per respondent) as those who would provide assistance in emergency situations. However, respondents shared emergency preparedness information with only 9% (M=1 member) and discussed disaster plans with only 16% (M=1.7 members). Respondents’ perceived preparedness to provide care in general was higher compared to their perceived preparedness to provide care in emergency situations (p<.001 for difference in means). Respondents with a greater number of assistance providers in their network reported higher caregiving preparedness in disaster situations (b=0.09, p=0.02) but not higher caregiving preparedness in general. Results suggest that caregivers may mobilize their support networks differently in the context of general caregiving versus caregiving in emergency situations. Efforts to increase discussions about disaster preparedness and management plans with support network members will be beneficial. Future research should focus on identifying strategies to strengthen caregiving support networks to be resilient in disaster situations.

